# Albany Hospital

**Published:** 1871-12

**Authors:** 


### Miscellaneous.

From the Albany Argus, Oct. 14, 1871.

### Albany Hospital.

We publish the following excellent advice, by Professor Thomas Hun, to the Governors of the Albany Hospital.

The following letters declining appointments have been addressed to the Governors:

ALBANY, Oct. 6, 1871.

*Geo. B. Steele, Esq., Sec'y, &c.:*

Dear Sir—I received last evening your note enclosing the copy of a resolution passed unanimously by the Governors of the Albany City Hospital.

This resolution appoints myself with other gentlemen to positions upon the surgical and medical staff of the institution

While it is my wish to perform all professionalobligations, and co-operate in earnest efforts that the hospital may enjoy full and unreserved confidence in the community, I do not think the resolution attains this purpose, or that my acceptanee of the position would promote the object which the Governors as well as myself, earnestly desire.

The resolution in no way changes the status which impelled my resignation, nor can I see in it the evidence of that spirit of radical reform in the administration which will ensure genuine and hearty co-operation between the Governnors and staff as well as permanent and assured prosperity to the institution.

I beg, therefore, you will convey to the gentlemen of the Board myregrets in feeling compelled to decline the appointment.

Respectfully yours,

S. OAKLEY VANDERPOEL.

ALBANY, Oct. 6, 1871.

*Geo. B. Steele, Esq.:*

Sir—I have received your communication informing me of the action of the Board of Governors of the Albany City Hospital, in appointing me a member of the medical staff of that institution.

I beg through you to offer to the Board of Governors my thanks for the honor they have seen fit to confer upon me, and regret that it is not possible for me to accept it.

The withdrawal of certain former members of the staff from all connection with the hospital, and the attempted expulsion of others, for no apparent reason except that they were displeasing to Dr. Armsby, renders it certain in my mind that, as you now propose to continue it, there can be no harmony or unanimity of action in the staff so long as Dr. Armsby is connected with it. Until I can see a probability of the existence of such harmony, I must decline serving on the hospital staff.

I was nominated over a year ago by the medical and surgical staff of the Albany Hospital to fill a vacancy occasioned by the death of Dr. Pomfret, and was rejected by the Board of Governors mainly through the personal efforts of Dr. Armsby; and I can see no reason why my presence on the staff should be more acceptable to him now than it would have been then.

Again by a resolution passed by you nearly two years ago, you declared that it was inexpedient for any physician attending at the Albany Hospital to be connected in a similar capacity with another institution. Therefore, in order to accept your offer I should be obliged to sever my connection with St. Peter's Hospital, which I am not prepared to do.

I am therefore compelled to decline the appointment conferred upon me.

Very respectfully yours,

EDWARD R. HUN.

---

*To the Governors of the Albany Hospital:*

Gentlemen—I have received from the secretary of your board a communication dated October 4, informing me that I had been appointed consulting physician of the hospital. I respectfully decline the appointment and feel it due to myself and respectful to you to state the reasons which induce me to do so.

The communication caused me great surprise, for in a conversation with Mr. Olcott and Mr. R. H. Pruyn, I had given the reasons why was unwilling to accept an appointment, and in a friendly but frank and unreserved manner had expressed to them my views as to the causes which had induced the unfortunate condition of the affairs of the hospital. If the views I expressed and the statements I made had been presented to your board, I think you would have withheld the action you have taken, and thus relieved me from an embarrassment, for I had no desire to appear publicly in this matter, or to give publicity to what I then said. But since notwithstanding the notice I gave, the appointment has been made and publicly announced, I now feel called on to break the silence I had imposed on myself, and to lay before your board and the public the statements and opinions expressed by me in the interview referred to. Without this explanation, my action in declining the appointment, coinciding with that of others similarly situated, might be misinterpreted by the public as indicating a spirit of hostility to the hospital and a determination to resist all offers of conciliation.

I stated to these gentlemen that in addition to my general disinclination to mingle in controversies and strife, I had an insuperable objection to occupy a position on a medical staff with Dr, Armsby; that an experience of many years satisfied me that he and I could not act harmoniously in the performance of hospital duties, and that beside this radical difference in our views, I was especially averse to an association with him in this hospital because of the anomalous position he occupies in it.

It is well known to all of you that during the last few years Dr. Armsby has assumed the entire control of the Hospital, and that the action of your Board has been steadily directed toward the support of such assumption. The superintendent and all subordinates have been selected by him, and have received their orders from him, and he has not hesitated to interfere with and overrule arrangements of his associates whenever it has seemed fit for him to do so. If a vacancy was to be filled in the medical staff, the question before you has been, not how well is the candidate qualified for the place, but how is he affected toward Dr. Armsby. Those of his associates who became obnoxious to him were marked for dismissal, and in all respects the Hospital has been managed with a view of sustaining Dr. Armsby. Remonstrances against this management on the part of a small minority of your Board or of members of the medical staff were met wlth the reply, "Dr. Armsby has rendered great service to the Hospital, and he must be sustained."

It must be remarked that "sustaining Dr. Armsby" did not mean protecting him against assaults, for no one thought of interfering with him in the performance of his duties, or of removing him from his position, but it meant his assumption to interfere in everything and to control everything.

I appeal confidently to you all, whether it is not literally true that no man, however eminent in his profession, or whatever promise of usefulness he might hold out, could within the last few years have received a medical appointment in the Hospital if he were obnoxious to Dr. Armsby.

Now it may be that your Board thought the services of the Doctor in procuring contributions were so important, or that his administrative abilities were so eminent, that it was best to maintain him in his position, but surely it cannot appear strange to you or to the public that his associates were unwilling to be kept in a position of inferiority to one who could justify his pretensions neither by superior weight of character nor professional standing.

Allow me to recall to you a few events in the recent history of the Hospital illustrating my assertions.

Dr. Robertson, by his energy in establishing an Eye and Ear Infirmary, secured from the State an appropriation of $4,000 which, by his connection with the Hospital, passed into its treasury. Far more than this, he placed at the service of the institution his eminent skill in his capacity, by which he added to its reputation and extended its usefulness. He offended Dr. Armsby and was removed.

Some others of the staff were not reckoned among the adherents of the Doctor, to effect whose removal a regulation was invented by which they were made incapable of holding office because they served in another hospital. Some of them disregarded it, and your Board felt ashamed to enforce it, but Drs. Vanderpoel and Mosher retired in disgust from an institution promulgating such a stupid and odious regulation.

Then a petition was got up by Dr. Armsby and signed by his adherents on the staff, asking, in effect, that the names of Drs. Boulware, Quackenbush and myself be dropped from the medical staff and other names substituted in their place, and your Board, ever ready to "sustain" the Doctor, expelled us. I do not bring this up as a personal grievance, for I had already ceased to take any active part in the affairs of the Hospital and would have resigned if requested, and besides this I have been informed by signers of the petition, and by prominent members of your Board that they did not know that I was affected by their action. But what a commentary is this on the manner in which every project arranged by the Doctor is so blindly carried out by his adherents on the staff, and even by your Board, that they do not find out till afterwards what they have done.

And so the Hospital has been gradually relieved from all the opponents of Dr. Armsby and none but his professed adherents now remain. No one of his subjects openly rebels against him, though it is said, some of them occasionally relieve themselves by abusing him behind his back. This is the *harmony* at which you have been arriving. (The silencing of all sounds but one does indeed prevent discord, but does not produce harmony, which consists in the accord of different sounds. Submission would better express your meaning.)

To crown all, at the election just held you have been confirmed in your authority, and thus are successful at every point. And yet as often happens, when men follow their own will, without regard to the rights and feelings of others, this series of successes has ended in what you now begin to find, is a failure. You do not feel satisfied with your own work and the public is even less satisfied than you are.

You have been so intent on securing harmony, as you call it, that you have lost sight of the fact that you were driving off your best men; for accord with Dr.. Armsby is not always the accompaniment of high professional standing. You are beginning to see that with one or two exceptions which it would be invidious to name, the strength of the profession is outside of the Hospital.

This may seem an ungracious thing to say, but I am engaged in a serious work and cannot let any false modesty or delicacy hinder me from saying that which you all know to be true and which is essential to the completion of my statement.

If my assertion is denied, then I ask, why these appointments have been offered to those who have so lately been driven off or expelled? What changes have occurred to make them more fit to be members of the staff now than then? Their places have been filled, and your staff is now more than sufficiently numerous. It counts enough, but does not weigh enough.

You did not think of this when in your eagerness for the removal of all that stood in your way, the heat and dust of the contest blinded you as to the tendency of your efforts. You were thinking then only of "sustaining Dr. Armsby." Now you find that you have to sustain not only him, but also the supports you have put under him, and begin to feel your burden.

In the hour of triumph you see that your victory has been barren.

Whose fault is it that the condition of the Hospital is not satisfactory? Those whom you have expelled have quietly retired, and have never attempted to embarrass its management. They have not even contested your resolution of expulsion which they are assured is legally invalid. They did make a supreme effort to restore a healthy life to the Hospital, and to relieve you from a painful responsibility, at the election just held, but they failed. They did their best and failed.

A good deal has been said about doctors quarrels, as if, in some way, they were the cause of the trouble. But what has a Board of Governors to do with doctors' quarrels? When you choose a doctor for your family, you select one who in your judgment is capable and faithful, and do not ask how he stands affected to this or that doctor. So in selecting doctors for the sick in the Hospital under your guardianship, your duty is equally clear; you have only to take the best men for the work to be done. Unfortunately you have been induced to attempt to secure the victory for one side of the combatants, and have come out of the fight with some scratches, as often happens to those who meddle in domestic quarrels.

Some of you now ask for co-operation to place the Hospital on a footing of more extended usefulness. It is one of the good traditions of our profession that none of its members has a right to refuse aid according to his ability, to a public charity which calls for it, and those whom you have driven out with insult are ready to co-operate with you—but on conditions. No hard conditions, but only the recognition of some elementary principles of which you have lost sight.

You are elected by the donors so to manage this charity as to carry out its true end, the relief of the sick and suffering. You were not elected to sustain or punish this or that Doctor, or to break down or strengthen this or that medical party.

Choose then from all the Physicians and surgeons of the city, those who have the confidence of the public, and hold out the best promise of usefulness to the sick. Let by-gones be by-gones, forget about doctors' quarrels, lay aside personal partialities, choose only with reference to the good of those who are placed under your guardianship; choose those whom you would choose for yourselves, and when chosen, sustain them in the performance of their duty, not one against the other, but altogether.

On these terms the co-operation of all good men is pledged to you.

Very respectfully,

THOMAS HUN.

ALBANY, October 4, 1871.

